# A novel mathematical model of protein-bound uremic toxin kinetics during hemodialysis

**DOI:** 10.1038/s41598-017-10981-z

**Published:** 2017-09-04

**Authors:** Vaibhav Maheshwari, Stephan Thijssen, Xia Tao, Doris Fuertinger, Franz Kappel, Peter Kotanko

**Affiliations:** 1 Renal Research Institute, New York, USA; 2 Institute for Mathematics and Scientific Computing, University of Graz, Graz, Austria; 3 Icahn School of Medicine at Mount Sinai, New York, USA

## Abstract

Protein-bound uremic toxins (PBUTs) are difficult to remove by conventional hemodialysis; a high degree of protein binding reduces the free fraction of toxins and decreases their diffusion across dialyzer membranes. Mechanistic understanding of PBUT kinetics can open new avenues to improve their dialytic removal. We developed a comprehensive model of PBUT kinetics that comprises: (1) a three-compartment patient model, (2) a dialyzer model. The model accounts for dynamic equilibrium between protein, toxin, and the protein-toxin complex. Calibrated and validated using clinical and experimental data from the literature, the model predicts key aspects of PBUT kinetics, including the free and bound concentration profiles for PBUTs and the effects of dialysate flow rate and dialyzer size on PBUT removal. Model simulations suggest that an increase in dialysate flow rate improves the reduction ratio (and removal) of strongly protein-bound toxins, namely, indoxyl sulfate and p-cresyl sulfate, while for weakly bound toxins, namely, indole-3-acetic acid and p-cresyl glucuronide, an increase in blood flow rate is advantageous. With improved dialyzer performance, removal of strongly bound PBUTs improves gradually, but marginally. The proposed model can be used for optimizing the dialysis regimen and for *in silico* testing of novel approaches to enhance removal of PBUTs.

## Introduction

Protein-bound uremic toxins (PBUTs) are inadequately removed during conventional hemodialysis (HD). A high degree of protein-binding reduces the free toxin concentration^1^, thereby diminishing the concentration gradient across the dialyzer membrane, the driving force for diffusive removal of toxins. In HD patients, a large number of PBUTs are found in excess, e.g. 3-carboxy-4-methyl-5-propyl-2-furanpropionate (CMPF; >99% protein-bound), hippuric acid (HA, ≈50% protein-bound), indole-3-acetic acid (IAA; ≈75% protein-bound), indoxyl sulfate (IS; ≈93% protein-bound), p-cresyl glucuronide (pCG; ≈15% protein-bound), and p-cresyl sulfate (pCS; ≈95% protein-bound)^2–4^. Several of these PBUTs mediate biological damage and are linked to adverse clinical outcomes^5^. Epidemiological research^6, 7^, PBUT-lowering studies^8^, and laboratory experiments^3, 9^, indicates that PBUTs contribute to the morbidity and mortality of patients with chronic kidney disease (CKD).

In HD patients, prototypical PBUTs, such as IS and pCS, have been found to be as much as 116-fold and 41-fold higher, respectively, than in age-matched healthy controls, while pre-dialysis concentrations of toxins with comparable molecular weight, such as urea and creatinine, were only 5- and 13-fold higher, respectively^10^. Of the factors contributing to the disproportionally higher levels of specific PBUTs, poor dialytic removal due to protein binding ranks highly. Unintuitively, for toxins with high albumin binding affinity, such as CMPF, post-dialysis serum concentration can in fact be higher than the pre-dialysis concentration, resulting in a negative reduction ratio^11^. Proposed approaches to enhance the removal of PBUTs include increasing the volume of blood processed by increasing the dialysis duration and/or dialysis frequency^4^, increasing the dialysate flow rate and the dialyzer mass transfer-area coefficient beyond standard dialysis practice^1, 12^. The added convective component of hemodiafiltration adds only little beyond the PBUT removal achieved in standard high-flux HD^13, 14^. Additional approaches to remove PBUTs also exist, notably (i) adsorption of toxins during dialysis on mixed-matrix membranes^15^ or on adsorbent zeolite silicalite^16^, (ii) maintaining the diffusive gradient across the dialyzer fiber by circulating albumin^17^ or activated charcoal in the dialysate^18^, (iii) displacing PBUTs from albumin using binding competitors to increase the free toxin fraction^19, 20^, and (iv) weakening the toxin affinity for albumin using a high-frequency electromagnetic field^21^.

A sound quantitative understanding of toxin kinetics during HD supports the identification of new avenues for enhanced toxin removal^22^. To this end, we developed a novel mathematical model providing a mechanistic understanding of PBUT kinetics which permits the simulation of effects exerted by various parameters on PBUT removal, such as dialysate flow rate, blood flow rate, dialyzer surface area, and free fraction of PBUTs. The proposed model accounts for compartmental distribution of PBUTs and the dynamic equilibrium between protein, toxin, and protein-toxin complex, both in the patient and dialyzer. The model was calibrated using patient data extracted from the literature^4^ and validated against *in vitro* ^1^ and *in vivo* findings^23^. Furthermore, the model predictions provide new insights into the dialytic removal of PBUTs.

## Results

### Model calibration

**Figure 1:**
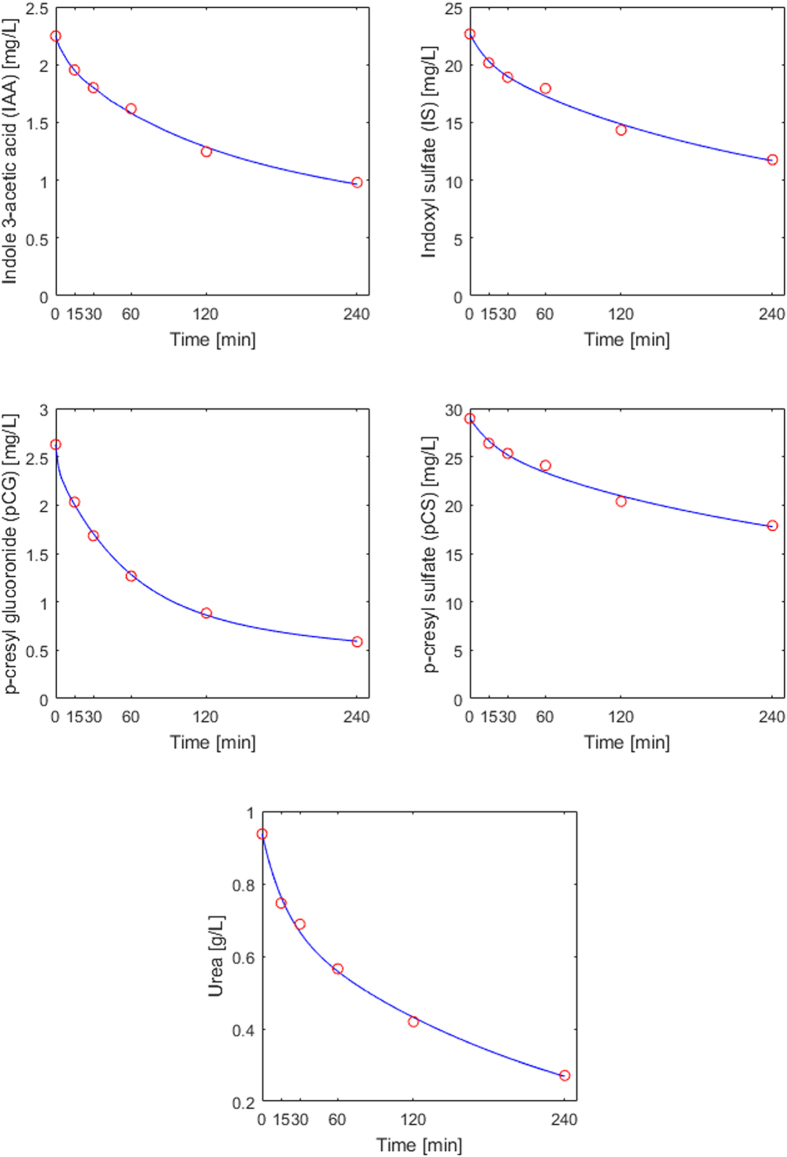
Intra-dialytic concentration profiles of protein-bound uremic toxins and urea. Circles depict data published by Eloot *et al*.^4^; lines represent model fit.

Representative patient data from a 10-patient cohort published by Eloot *et al*. were used for model calibration^4^; patients’ demographics are presented in Table 1. Table 2 shows the degrees of protein binding used to calibrate the model for IAA, IS, pCG, and pCS^4^. Initial plasma volume (*V*_pl0_), initial extracellular fluid volume (*V*_*ex0*_), intracellular fluid volume (*V*_*ic*_), the toxin mass transfer coefficient between plasma and the interstitial compartment (*K*_*ip*,*T*_), the toxin mass transfer coefficient between intracellular and the interstitial compartment (*K*_*ic*,*T*_), and the association constant (*k*_1_) for the dynamic equilibrium $P+T\,\rightleftharpoons PT$ were estimated using toxins’ time course data^4^. Table 3 shows the calculated toxin generation rate and resulting parameter estimates. Figure 1 shows the intradialytic time courses for the four PBUTs and urea^4^, with respective model fits. The calibration yielded good fit to these literature-reported patient data for all investigated solutes (per toxin, relative error across all samples is 2%).

**Table 1:** Demographics and clinical characteristics of HD patients(s) used in model calibration and validation. In model calibration, one patient data was used.

N	10 (8 men)
Age	69 ± 12 years
Hematocrit	37.2 ± 4.2%
Weight	75 [69.9; 82.6] kg
BMI	28 [25; 28] kg/m^2^
Renal function	2.6 [0.0; 4.1] mL/min

Data is presented as mean ± s.t.d. as well as Median [25^th^ percentile; 75^th^ percentile].Information obtained from Eloot *et al*.^4^ and Deltombe *et al*.^23^.

**Table 2:** Degree of protein binding reported by Eloot *et al*.^4^. The values represent averages of 10 hemodialysis patients.

Toxin	Protein-binding (%)
Indole-3-acetic acid (IAA)	73
Indoxyl sulfate (IS)	93
p-cresyl glucuronide (pCG)	13
p-cresyl sulfate (pCS)	95
Urea	—

**Table 3:** Generation rates and parameter estimates for PBUTs and urea using data extracted from Eloot *et al*.^4^.

Toxin	*G* (mg·min^−1^)	*K*_*ip*,*T*_ (mL·min^−1^)	*K*_*ie*,*T*_ (mL·min^−1^)	*k*_1_ (M^−1^·min^−1^)	*V*_*pl0*_ (L)	*V*_*ex0*_ (L)	*V*_*ic*_ (L)
IAA	0.0053	1366	160	1.5 × 10^3^			
IS	0.0285	1210	103	3.6 × 10^7^			
pCG	0.0136	1148	67	1.9 × 10^6^	2.4	12.9	25.9
pCS	0.0277	1060	98	4.0 × 10^7^			
Urea	8.17	—	363	—			

IAA – Indole-3-acetic acid, IS – Indoxyl sulfate, pCG – p-cresyl glucuronide, pCS – p-cresyl sulfate; *G* – Generation rate; *K*_*ip*,*T*_ – Toxin mass transfer coefficient between plasma and interstitial compartments; *K*_*ie*,*T*_ – Toxin mass transfer coefficient between interstitial and intracellular compartments; *K*_1_ – Association constant in protein-toxin dynamic equilibrium; *V*_*pl0*_ – Initial plasma volume; *V*_*ex0*_ – Initial extracellular fluid volume; *V*_*ic*_ – Intracellular fluid volume.

### Model validation

The model was validated using data from published reports of PBUT kinetics. Specifically, we investigated the quality of the model predictions within the following domains:

#### *Change in protein binding during HD*

**Figure 2:**
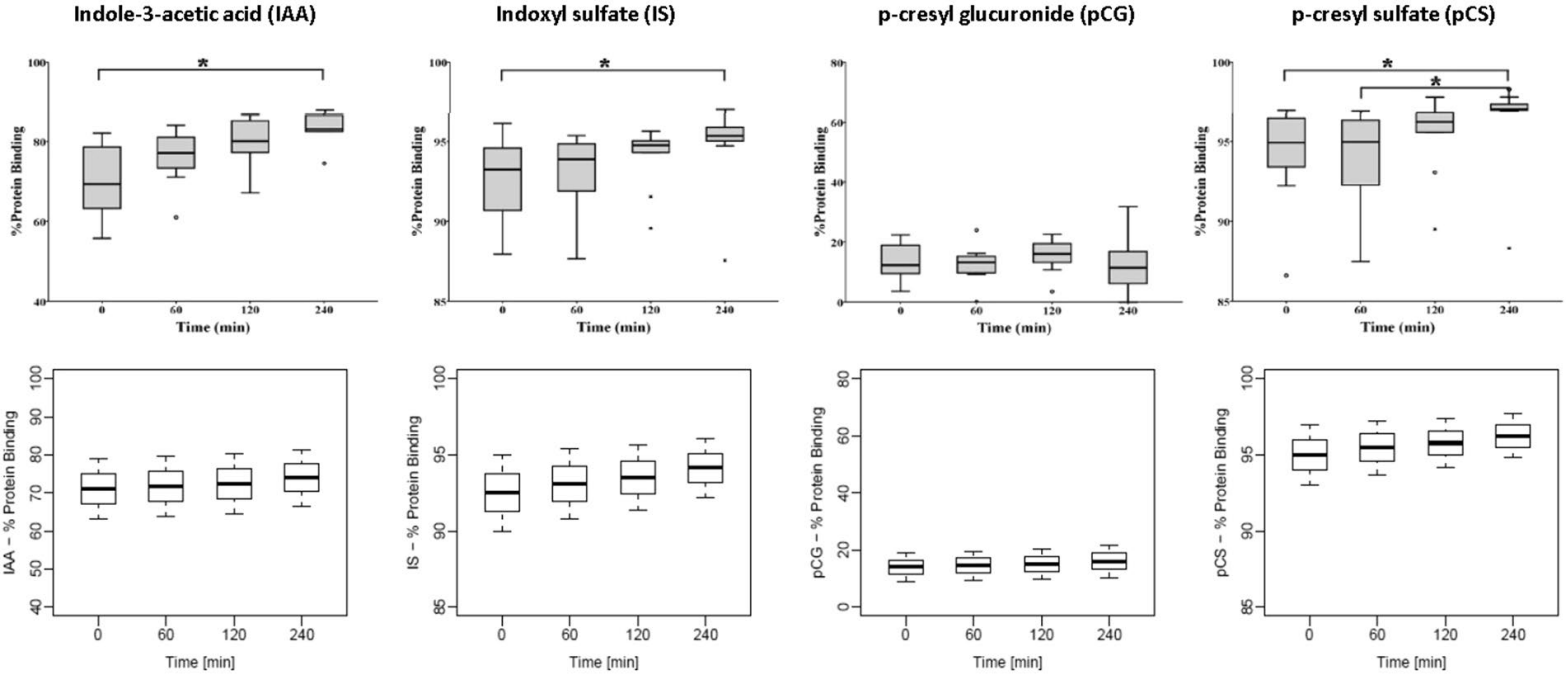
Percentage protein binding at different time points during the course of dialysis; left-to-right for indole-3-acetic acid (IAA), indoxyl sulfate (IS), p-cresyl glucuronide (pCG), and p-cresyl sulfate (pCS). Top row corresponds to *in vivo* data from 10 HD patients^23^ and bottom row shows model simulations.

With increasing PBUT concentrations, available albumin binding sites become progressively saturated, and the free fraction of these toxins increases; this is a well-documented phenomenon in CKD patients^24, 25^. Conversely, as PBUT concentrations drop during HD – and the ratio of albumin binding sites to PBUT molecules increases – the degree of protein binding of these toxins is expected to increase. Deltombe *et al*. published *in vivo* data on this phenomenon^23^. Figure 2 compares the intradialytic change in protein binding reported by Deltombe *et al*. to that observed in our model simulations for IAA, IS, pCG, and pCS. Table 4 presents the initial toxin concentrations and ranges of bound fractions used in our simulations.

**Table 4:** Initial concentration and range of bound fractions variation used in model simulations.

Toxin	Initial concentration (mg/L)^4^	Range of initial bound fraction^23^
IAA	2.1	63–80%
IS	15.1	90–95%
pCG	3.5	9–19%
pCS	30.6	93–97%

IAA – Indole-3-acetic acid, IS – Indoxyl sulfate, pCG – p-cresyl glucuronide, pCS – p-cresyl sulfate.

#### *Change in protein binding across the dialyzer fiber*

**Figure 3:**
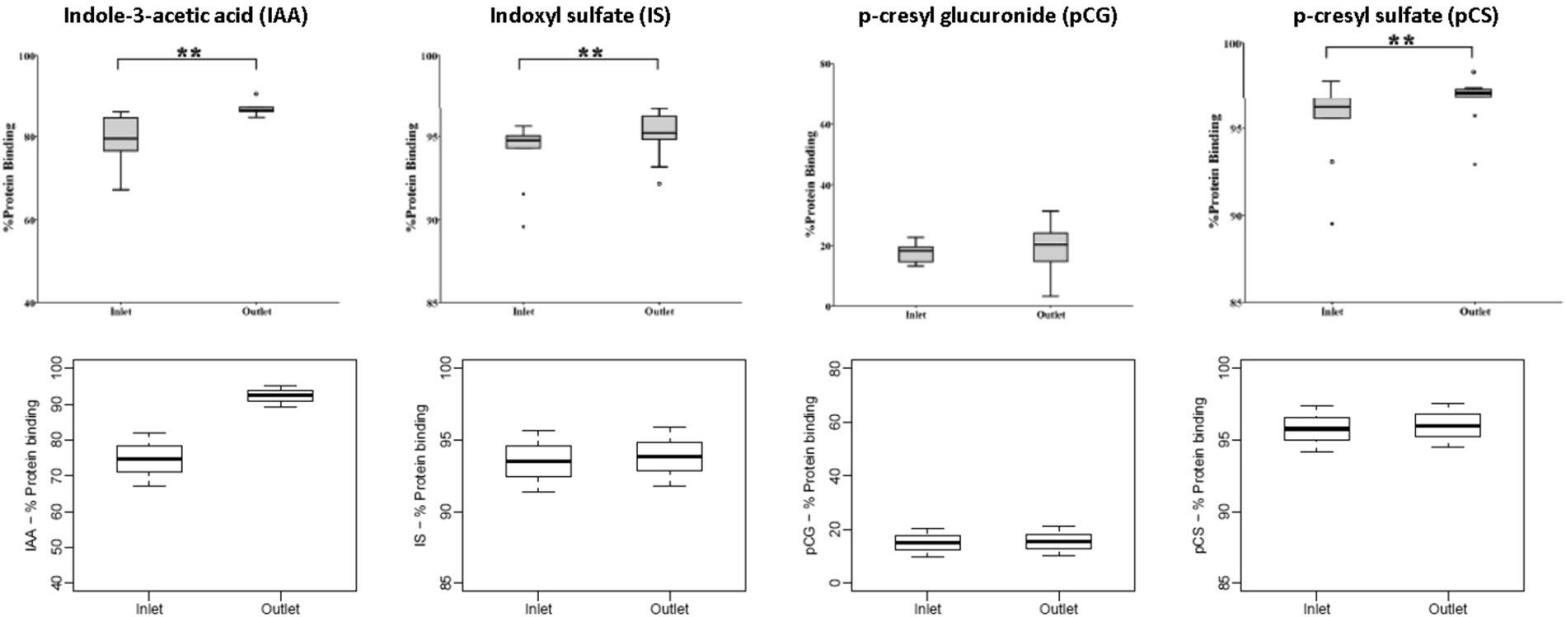
Percentage protein binding at the dialyzer blood inlet *versus* blood outlet after 120 min since dialysis start; left-to-right for indole-3-acetic acid (IAA), indoxyl sulfate (IS), p-cresyl glucuronide (pCG), and p-cresyl sulfate (pCS). Top row corresponds to *in vivo* data from 10 HD patients^23^ and bottom row shows model simulations.

Analogous to the effect of PBUT removal during the course of HD on the degree of protein binding described above, the bound PBUT fraction also increases along the dialyzer, from blood inlet to outlet^23^. Figure 3 compares *in vivo* results on this phenomena in 10 patients^23^ with our model simulations. The model accurately predicts the rise in protein binding from blood inlet to blood outlet.

#### *Intradialytic time-course of reduction ratios for free and total PBUT concentration*

**Figure 4:**
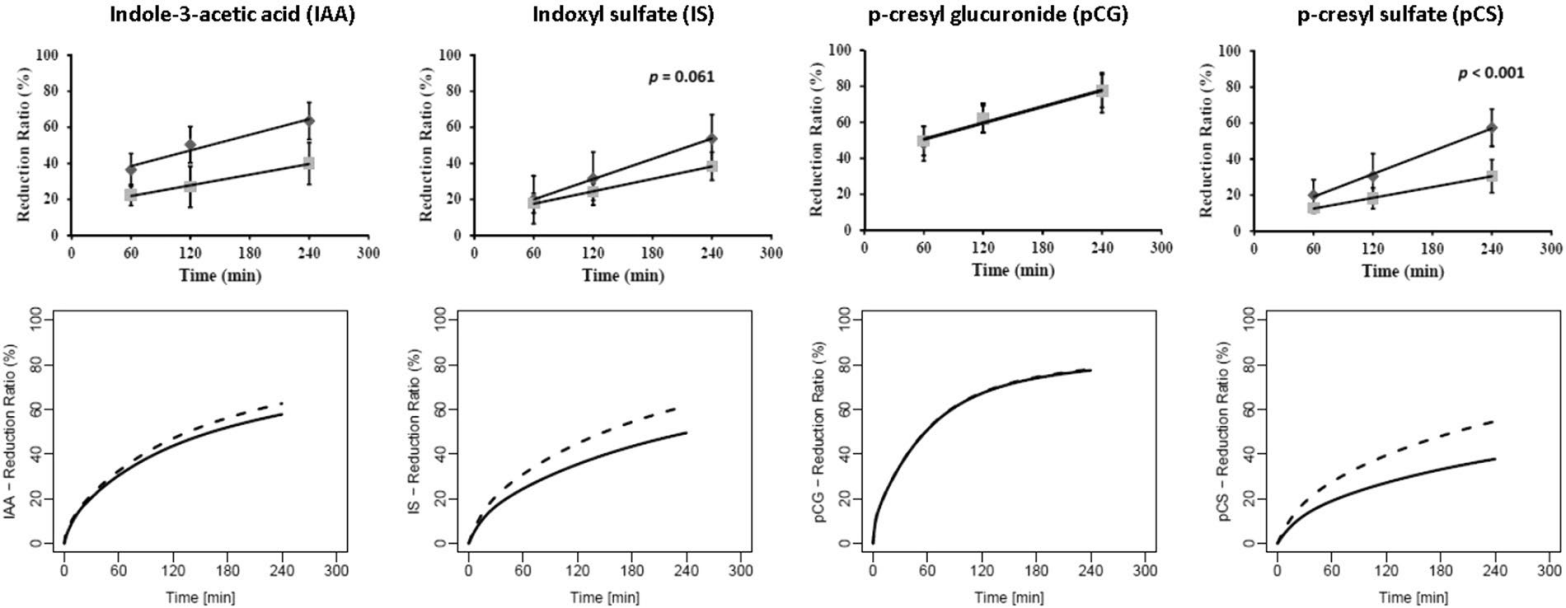
Reduction ratio for total () and free () toxin concentration at different time points during an HD session; left-to-right for indole-3-acetic acid (IAA), indoxyl sulfate (IS), p-cresyl glucuronide (pCG), and p-cresyl sulfate (pCS). Top row corresponds to *in vivo* data from 10 HD patients^23^ and bottom row shows model simulations (solid line for total toxin concentration and dashed line for free toxin concentration).

Figure 4 shows the intradialytic time-courses of reduction ratios (RRs) for the free and total concentrations of IAA, IS, pCG, and pCS. The top row shows *in vivo* observations^23^, the bottom row our model simulations. Here, too, the model captures the kinetics well, both qualitatively and quantitatively.

#### *Effect of dialyzer surface area and dialysate flow rate on PBUT removal*

**Figure 5:**
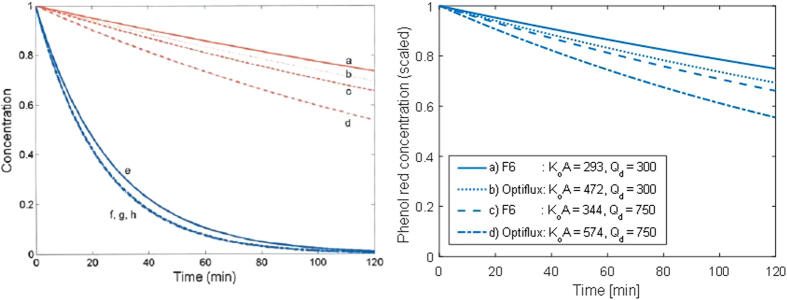
Effect of varying ${{\mathscr{K}}}_{o}{\mathscr{A}}$ and *Q*_d_ on removal of phenol red. The left panel shows model predictions by Meyer *et al*.^1^, which closely matched their *in vitro* results obtained in bench dialysis experiments using artificial plasma (figure reproduced with permission). The blue lines (**e**–**h**) describe urea removal and are not part of this discussion. The right panel shows results obtained with our mathematical model by simulating the scenarios studied by Meyer *et al*.^1^. Line styles and labeling correspond to the same scenarios, which are the following: (**a**) F6, *Q*_d_ 300 ml/min, *Cl*_PR_ 11 mL/min; (**b**) Optiflux F200NR, *Q*_d_ 300 mL/min, *Cl*_PR_ 14 mL/min; (**c**) F6, *Q*_d_ 750 mL/min, *Cl*_PR_ 16 mL/min; (**d**) Optiflux F200NR, *Q*_d_ 750 mL/min, *Cl*_PR_ 23 mL/min. The ${{\mathscr{K}}}_{o}{\mathscr{A}}$ values presented in the right panel are calculated using phenol red clearance values and Meyer *et al*. clearance expression for PBUTs (reproduced in the Methods section)^1^.

Meyer *et al*. investigated the effects of dialysate flow rate and dialyzer mass transfer-area coefficient $({{\mathscr{K}}}_{o}{\mathscr{A}})$ on the clearance of phenol red (≈94% albumin-bound) in an *in vitro* dialysis using artificial plasma^1^. They studied two dialyzers (Fresenius F6 and Optiflux F200NR), each at two dialysate flow rates (300 and 750 mL/min) to cover different combinations of dialysate flow rate and dialyzer ${{\mathscr{K}}}_{o}{\mathscr{A}}$. Meyer *et al*. then developed a mathematical model describing the removal of strongly bound PBUTs, and confirmed that their model accurately predicted the effects of varying ${{\mathscr{K}}}_{o}{\mathscr{A}}$ and dialysate flow rate. We utilized the reported phenol red clearance data to calculate the corresponding ${{\mathscr{K}}}_{o}{\mathscr{A}}$ values^1^ and applied our model to simulate the *in vitro* scenarios studied by Meyer *et al*.^1^. As shown in Figure 5, our model predictions closely resemble Meyer’s experimental findings.

### Model predictions

**Figure 6:**
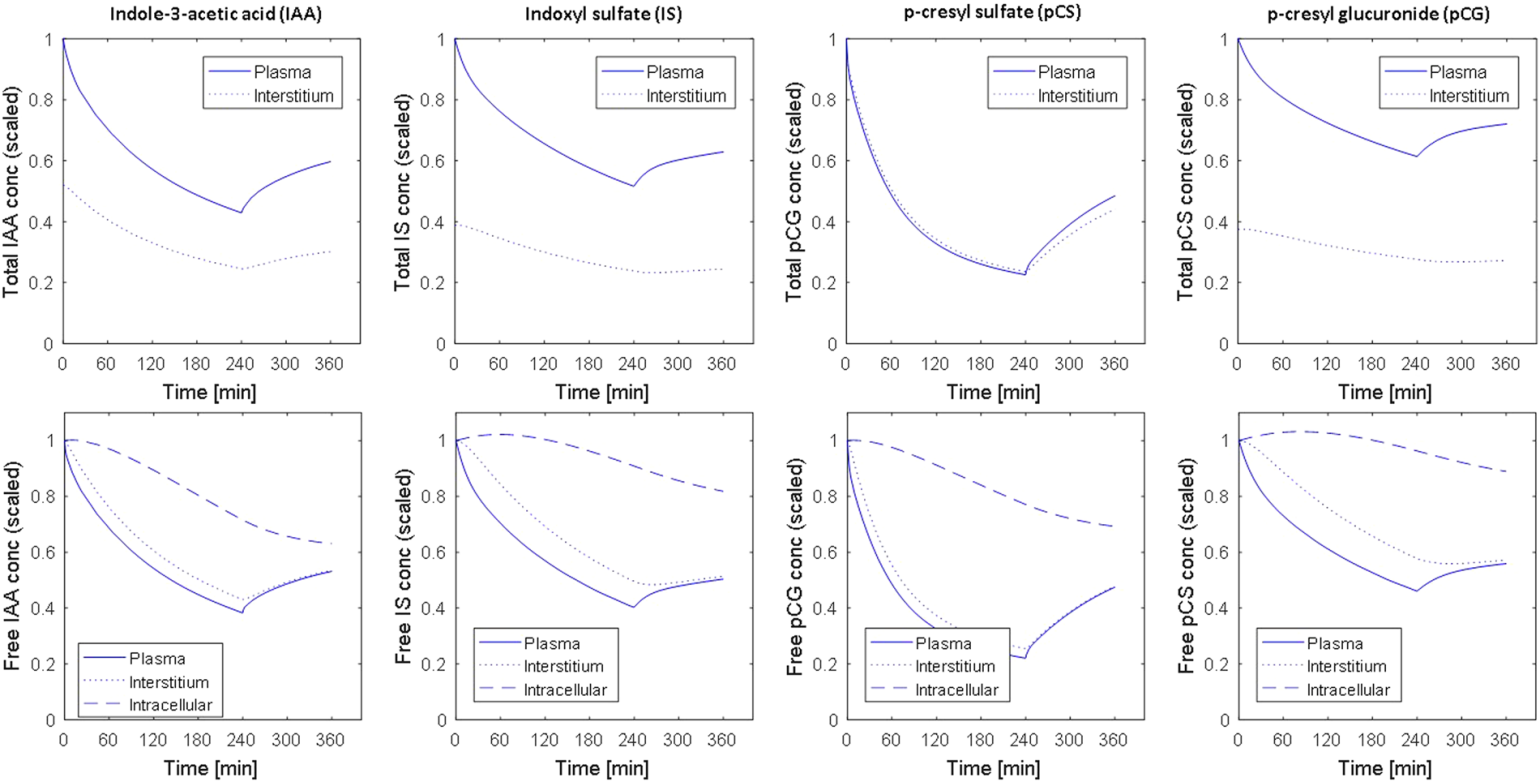
Total (top row) and free (bottom row) protein-bound uremic toxin concentrations during and after dialysis; left-to-right for indole-3-acetic acid (IAA), indoxyl sulfate (IS), p-cresyl glucuronide (pCG), and p-cresyl sulfate (pCS). Concentrations are scaled in relation to pre-dialysis plasma concentration. Solid lines denote plasma concentrations, dotted lines interstitial concentrations, and dashed lines free intracellular concentrations. Note that free and total toxin concentrations in the intracellular compartment are identical, as there is no intracellular albumin. Therefore, intracellular concentrations are only shown in the bottom plots.

**Figure 7:**
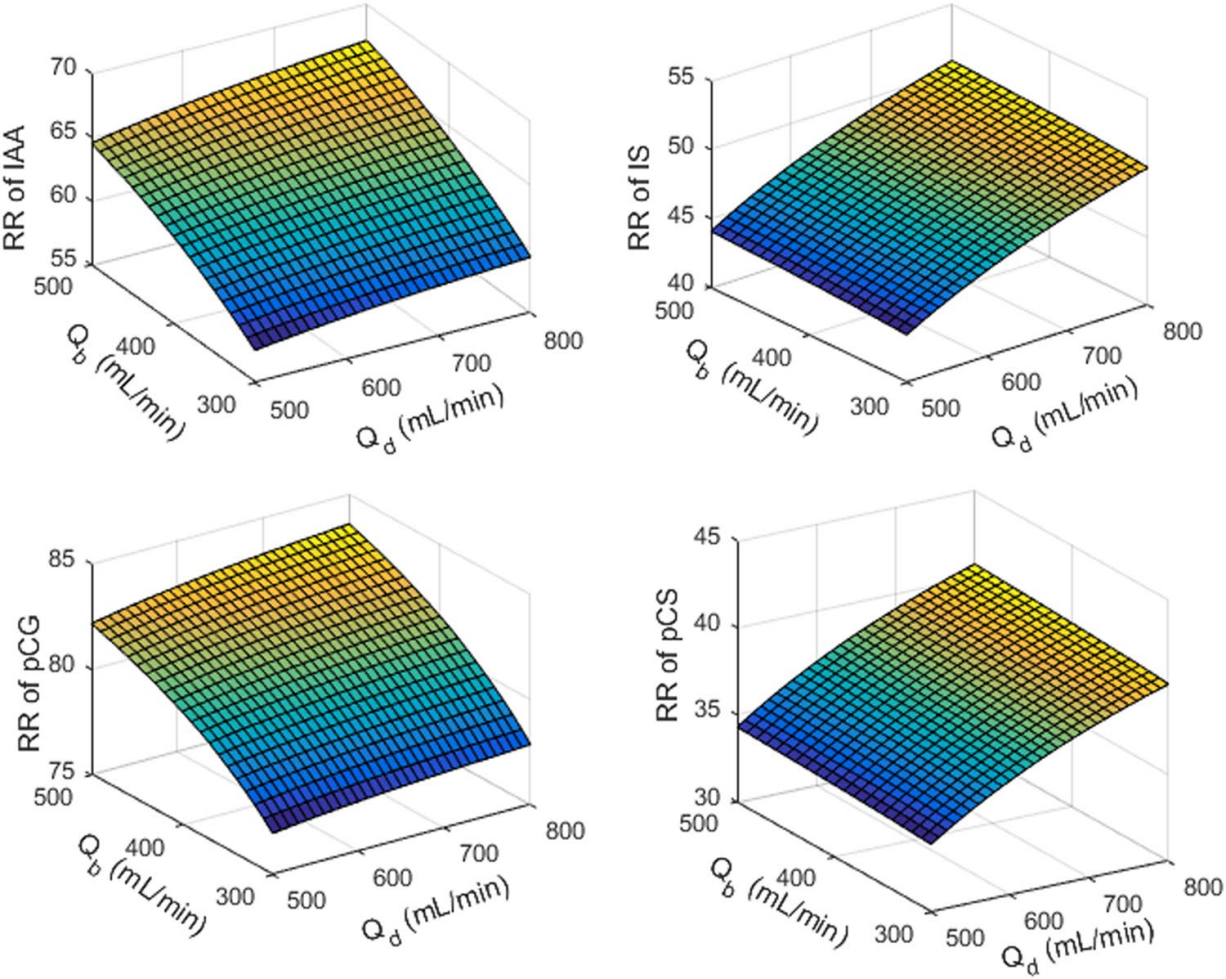
Effect of blood flow rate (*Q*_b_) and dialysate flow rate (*Q*_d_) on reduction ratio (RR) calculated for total protein-bound uremic toxin concentration. Clockwise from top left: indole-3-acetic acid (IAA), indoxyl sulfate (IS), p-cresyl sulfate (pCS), and p-cresyl glucuronide (pCG). The simulations were performed for F180NR dialyzer with *Q*_b_ and *Q*_d_ were 300 and 700 mL/min, respectively; ultrafiltration volume 2 L. Initial toxin concentrations were same as in model calibration or Figure 1 and corresponding model parameters were taken from Table 3.

Our model also predicts the distribution of PBUTs (both free and bound) in the major physiological compartments, as well as the post-dialytic rebound (Figure 6 for IAA, IS, pCG, and pCS). Additionally, the effect of blood and dialysate flow rate on the RRs of individual PBUTs is shown in Figu﻿re 7. For strongly bound PBUTs (IS and pCS), an increase in dialysate flow rate has a much more pronounced effect on RR than an increase in blood flow (Figure 7, right column). For moderately/weakly bound PBUTs (IAA and pCG), on the other hand, an increase in blood flow rate yields the higher return (Figure 7, left column).

## Discussion

PBUTs are known to exert several deleterious effects in dialysis patients^2, 5, 26, 27^. It is, therefore, reasonable to assume that enhanced removal of these toxins might translate into better patient outcomes^5, 28^. Systematic development of methods to improve dialytic removal of PBUTs requires a sound quantitative understanding of the interplay between the underlying mechanisms that limit their removal. These interplay can be understood via toxin kinetic modeling^22^, as previously demonstrated in the case of non-protein-bound toxins^29–31^.

To the best of our knowledge, only two approaches of mathematical modeling have been presented for PBUTs. Meyer *et al*. were the first to model the kinetics of strongly bound PBUTs^1^. In their model, the expression for dialytic clearance of PBUTs is adjusted by the free toxin fraction (*f*). Their model predicted that increasing the dialysate flow with concomitant increase in dialyzer surface area would improve PBUT removal; the findings were tested *in vitro* using phenol red, a substance with very high affinity towards albumin^1^, and *in vivo* in 6 HD patients for IS, pCS, kynurenic acid, and hippurate^12^. The model assumed a constant free fraction along the dialyzer fibers – an assumption which is questionable based on first principles and is at variance with *in vivo* data showing that the bound PBUT fraction increases along the dialyzer fiber (Figure 3)^23^. Additionally, the model by Meyer *et al*. did not account for physiological distribution of PBUTs. Notwithstanding these limitations, this first modeling approach recommended maneuvers to improve removal of strongly bound PBUTs.

The second modeling approach of PBUT kinetics, a conventional two-compartment model lacked important fundamental characteristics of PBUTs that impact their dialytic removal, namely, strong binding to albumin, unequal concentration distribution in physiological compartments, and dialytic removal driven by free rather than total concentration^4^. Our model captures these important characteristics missing in the aforementioned two models, thereby contributing a more comprehensive and faithful representation of PBUT kinetics.

While previous modeling approaches assumed that dialytic clearance is driven by total plasma concentration of PBUT^1, 4^, in reality, the toxin flux across the dialyzer membrane is driven by the free toxin concentration gradient between plasma and dialysate. Mechanistically, PBUT removal occurs via diffusive transfer of free toxin from plasma into the dialysate. As soon as the free toxin is removed from the blood side, the protein-toxin complex assumes a new equilibrium. This dynamic equilibrium is governed by Le Châtelier’s principle, which states that a chemical equilibrium will readjust itself to changes in concentration, pressure, temperature, and/or volume^32, 33^. The present modeling approach considers the dynamic equilibrium between protein, toxin, and the protein-toxin complex both in the patient and dialyzer. We, therefore, proposed a coupled model of the patient and dialyzer system where PBUT exchange in the patient is represented by a three-compartment model comprising plasma, interstitial, and intracellular compartment, while the dialyzer model describes the spatiotemporal changes of protein, toxin, and protein-toxin complex concentrations along the dialyzer.

We calibrated our model using patient data reported by Eloot *et al*.^4^. In contrast to the conventional two-compartmental modeling approach, where toxin distribution volume is estimated separately for each toxin^4^, we adopted a more physiological approach where we assume that in a given patient the volumes of plasma, interstitial, and intracellular compartments are independent of the PBUT modeled. This approach also reduces the number of unknown parameters. Unlike non-protein bound toxins, total initial concentrations of PBUTs differ in between the plasma and interstitial pool due to unequal molar distribution of albumin. This is accounted for by modeling both the plasma and the interstitial compartment for PBUTs; however, pre-dialysis free concentration is assumed to be in equilibrium between plasma, interstitial, and intracellular compartments.

On the other hand, as urea is a non-protein-bound solute, separate plasma and interstitial compartments were not considered for urea, and a conventional two-compartment model representation of urea kinetics was used in tandem with the proposed three-compartmental PBUT patient model. The mass transfer rates *K*_*ip*,*T*_ and *K*_*ic*,*T*_, and the association constant *k*_1_ were estimated independently for each PBUT, while for urea only *K*_*ic*,*T*_ was estimated. For PBUTs, *K*_*ip*,*T*_ (1060 to 1366 mL/min) is much larger than *K*_*ic*,*T*_ (67 to 160 mL/min), with about an order of magnitude difference for individual PBUTs (Table 3). These parameter estimates are physiological, because capillary endothelium separating the plasma and interstitial compartment is much more permeable to small solutes than cell membranes, thus presenting a lower resistance to diffusion^34^. Conventional two-pool models assume that equilibration between plasma and the interstitial pool is instantaneous – this is equivalent to assuming an infinite inter-compartmental mass transfer coefficient. The large estimate of *K*_*ip*,*T*_ obtained here appears to justify this assumption that is made for many non-protein bound toxins^35^. On the other hand, the *K*_*ic*,*T*_ values for all toxins are of similar magnitude, while the *K*_*ic*,*T*_ for urea (363 mL/min) is higher than *K*_*ic*,*T*_ for the PBUTs (67 to 160 mL/min). This higher *K*_*ic*,*T*_ for urea may be due to (1) urea being smaller in size than the other toxins considered here, thus experiencing smaller diffusive resistance by cellular membranes, and (2) the fact that urea transport from the intracellular space is not only driven by passive diffusion but also augmented by selective urea transporters^36^.

This calibrated model not only provides meaningful physiological estimates of model parameters but also excellent prediction of existing *in vitro* and *in vivo* data^1, 23^. Deltombe *et al*. observed that during dialysis, the protein-bound fraction of PBUTs increases^23^. Our calibrated model is able to predict this phenomenon closely (Figure 2). This can be explained by the fact that free toxin is dialyzed during dialysis – resulting in less solute competing for available binding sites, which increases the possibility of binding. Since protein is non-dialyzable, its concentration increases continuously due to ultrafiltration. This continuous increase in protein concentration (surrogate of binding sites) will shift the dynamic equilibrium towards formation of protein-toxin complex and thus increase in the fraction of bound toxin.

With removal of free toxin and availability of more binding sites, the decrease in total and protein-bound toxin concentrations will decelerate. The same rationale also explains the increase in bound toxin fraction between dialyzer inlet and outlet (Figure 3) and the higher RR for free compared to total toxin concentration (Figure 4). With stronger toxin affinity towards albumin, as in the case of IS or pCS, one may notice generally lower RRs. Also, with higher binding affinity between albumin and toxin, i.e. with a lower free fraction available for dialysis, the gap between the RRs of free and total concentration widens over the course of HD, with that gap increasing in the order pCG < IAA < IS < pCS (Figure 4). The RRs seen in our model simulations are typical for this class of toxins^11, 37^. In the above three Figures 2, 3 and 4, the *in vivo* data (top rows in the corresponding figures) were extracted from Deltombe *et al*.^23^. In their paper, only population level data are presented, and no individual patient data are shown. Hence, we provide a qualitative comparison on a population level only. This is one of the limitations of our work.

Notably, the RR of pCG (78%) is higher than that of urea (71%). Based on these RRs, one should not deduce that intra-dialytic removal of pCG is superior to urea; the absolute solute removal is greater for urea than any of the simulated PBUTs because of its higher concentration. A better metric for comparison is the relative removal of individual toxins, i.e. the percentage of total initial toxin load removed in a single HD session. Using the toxin concentration data and estimated distribution volumes, we can calculate the pre- and post-dialysis toxin loads. Based on this, the relative removal of urea and pCG is 69% and 45%, respectively. The higher relative removal of urea can be attributed to its higher *K*_*ic*,*T*_, which replenishes the plasma compartment with urea at a much faster rate, resulting in higher plasma concentration.

On the other hand, pCG is efficiently cleared from plasma, but poorly replenished from the intracellular pool (Figure 6, column 3) because of a much lower *K*_*ic*,*T*_ (Table 3). This lower *K*_*ic*,*T*_ and continuous dialytic removal results in a lower intra-dialytic pCG concentration and higher RR. It is, however, not the lower *K*_*ic*,*T*_ alone that lowers the intra-dialytic concentration of pCG; if its *K*_*ic*,*T*_ is artificially increased to the same level as *K*_*ic*,*T*_ urea, the relative removal increases from 45% to 59%, still well below the relative removal of urea (69%). This suggests that protein-binding combined with lower mass transfer rate (*K*_ic,T_) from the intracellular to the interstitial compartment results in its poor removal. Plasma pCG concentration rebounds sharply after HD – a reflection of the wide gap between plasma and interstitial concentrations on one hand and intracellular concentration on the other (Figure 6, column 3). *In vivo* studies are needed to corroborate or reject these model predictions.

We were also able to capture the effect of increasing dialysate flow rate (*Q*_d_) and dialyzer surface area $({{\mathscr{K}}}_{o}{\mathscr{A}})$, as in Meyer *et al*.^1^. Furthermore, independent effects of blood and dialysate flow on the RRs of PBUTs are also illustrated. For strongly bound PBUTs (IS and pCS), our simulations suggest that an increase in blood flow rate (*Q*_b_) is not as effective as an increase in *Q*_d_ (Figure 7). On the other hand, for weakly bound toxins, the opposite is true and an increase in *Q*_b_ is much more effective than an increase in *Q*_d_ (Figure 7). These findings align with literature evidence showing a negligible effect of *Q*_d_ on dialysis dose *Kt/V*_urea_^38^, while significant increases in *Kt/V*_urea_ occur with increases in *Q*_b_^39^. A plausible physical explanation to this aspect is as follows: In the case of weakly bound toxins (IAA and pCG) free toxin concentration is high in blood/plasma. When this free toxin laden blood passes through dialyzer, a large fraction of free toxin is removed in a single pass. Increasing the blood flow rate increases the rate of blood circulations through the dialyzer and as such the solute removal across the dialyzer. However, in the case of IS and pCS, owing to strong protein-binding, the dialyzer outlet concentrations are reduced only slightly. Mixing of this dialyzer outlet blood with blood in the systemic circulation will result in only a very small decrease in their concentrations. Hence, increasing the blood flow rate (i.e. reducing blood recirculation time) will have an only minor effect on the RR of strongly bound toxins. On the other hand, increase in dialysate flow will increase the concentration gradient between blood and dialysate, resulting in higher solute removal. However, for weakly bound toxins, the RR magnitude is high irrespective of dialysate flow; increasing *Q*_d_ only marginally increases their RR. The dialysate flow effect is more visible for IS and pCS. Additional details describing the effects of blood and dialysate flow rate on PBUT removal are provided in the Supplemental Material to this manuscript. In summary, increase in *Q*_d_ and *Q*_b_ can improve the removal of PBUTs, but this improvement is marginal. Also, *Q*_b_ cannot be increased beyond 500 mL/min. Furthermore, this improvement is not linear – with further increase in *Q*_d_, the improvement in RR will decrease. New methods for improving the dialytic removal of PBUT, such as binding competition^20^, albumin dialysis^17^, and application of electromagnetic fields^21^ should be explored; this model can offer a critical perspective into the efficacy of these novel approaches for PBUT removal.

In conclusion, the proposed mathematical model offers a more comprehensive quantitative understanding of PBUT kinetics during HD. Model simulations corroborate the notion that high protein-binding plays a major role in inhibiting the removal of strongly bound PBUTs^2, 28^. Once free toxin is removed, only then the protein-bound fraction will dissociate, i.e. a smaller diffusive gradient due to protein-binding is the limiting factor in the removal of strongly bound PBUTs. For weakly bound PBUTs, a significant amount of toxin might be present in the intracellular compartment, and the additional factor of inter-compartmental resistance may become prominent and delay toxin transfer from the intracellular to the interstitial and plasma compartments. One may conclude that the kinetics of PBUTs significantly differ from non-protein bound toxins; nevertheless an analogy of the former can be made with the latter from a kinetics point-of-view – removal of strongly bound PBUTs is primarily restricted by a smaller diffusive gradient across the dialyzer membrane while removal of non-PBUTs is primarily restricted by inter-compartmental resistance^29, 40^. Moving forward, the combined use of mathematical modeling and clinical studies is warranted to guide the development of technologies resulting in an enhanced dialytic removal of PBUTs.

## Materials and Methods

We developed a multi-compartment patient model and a dialyzer model to describe the PBUT kinetics. We considered IAA, IS, pCG, and pCS as model PBUTs. We also used a two-compartment model for urea kinetic in order to compare it with PBUT kinetics and for parameter estimation. Description of all symbols appearing in model equations can be found in the Table 5.

**Table 5:** Nomenclature with description of symbols, corresponding units and values (only for constants).

Symbol	Description	Units	Value
*α*	Fraction of extracellular fluid volume in the interstitial compartment	—	—
*A*	Blood flow area of fiber (inner cross-sectional area of fiber)	m^2^	3.5 × 10^−8^
*A*_d_	Effective flow area for dialysate around a fiber (annulus space between fibers)	m^2^	4.06 × 10^−8^
*C*_*p/d*_	Concentration in plasma/dialysate side stream (*C* ϵ [*T*, *PT*, *P*])	M	—
*D*_*h*_	Dialyzer housing diameter	m	0.04
*f*	Toxin free fraction	—	—
*G*	Toxin generation rate	mg·min^−1^	—
*k*_1_	Association constant in P, T, and PT equilibrium	M^−1^ min^−1^	—
*k*_2_	Dissociation constant in P, T, and PT equilibrium	min^−1^	—
*K*_*A*,*toxin*_	Equilibrium association constant for individual PBUTs	M^−1^	—
*K*_*D*_	Conventional dialyzer clearance (urea)	mL·min^−1^	224
*K*_*ip*,*T*_	Free toxin mass transfer coefficient between interstitial pool and plasma	mL·min^−1^	—
*K*_*ic*,*T*_	Toxin mass transfer coefficient between intracellular and interstitial pool (for urea – between intracellular and extracellular)	mL·min^−1^	—
${{\mathscr{K}}}_{o}{\mathscr{A}}$	Dialyzer mass transfer-area coefficient	mL·min^−1^	600
*L*	Length of fiber	m	0.23
*N*	Number of fibers in dialyzer housing	—	12,300
*Pe*	Péclet number	—	0.0139
*P*_*pl*/*is*_	Protein (without toxin) concentration in plasma/interstitial compartment	M	—
*P*_*out*_	Protein (without toxin) concentration at dialyzer exit	M	—
*PT*_*pl*/*is*_	Protein-toxin complex concentration in plasma/interstitial compartment	M	—
*PT*_*out*_	Protein-toxin complex concentration at dialyzer exit	M	—
*Q*_*b/d/p*_	Blood/dialysate/plasma flow rate	mL·min^−1^	300/700/189
*q*_*pi/di*_	Inlet plasma/dialysate flow rate in/around single fiber	mL·min^−1^	*Q*_*b/p*/*d*_ */N*
*q*_*p/d*_	Plasma/dialysate flow rate at position *x* inside/around hollow fiber	mL·min^−1^	—
*Q*_*uf*_	Ultrafiltration rate	mL·min^−1^	8.3
*r*_*f*_	Hollow fiber inner radius	m	105 × 10^−6^
*t*_*f*_	Thickness of hollow fiber membrane	m	35 × 10^−6^
$\bar{T}$	Toxin concentration inside the membrane dependent on blood and dialysate side toxin concentration	M	—
*T*_*pl/is/ic*_	Free toxin concentration in plasma/interstitial/intracellular compartment	M	—
*T*_*out*_	Free toxin concentration at dialyzer exit	M	—
*V*_*pl*/*is*/*ex*/*ic*_	Plasma/Interstitial/extracellular/intracellular fluid volume	L	—
*V*_*pl0*/*ex0*_	Initial plasma/extracellular distribution volume	L	—

### Mathematical Model

#### (A) *Patient model*

We developed a three-compartmental patient model comprising plasma, interstitial, and intracellular compartments, with protein-bound fraction distributed in plasma and interstitial, and free fraction distributed in all three compartments. Albumin is the major binding protein of prototypical PBUTs such as IS and pCS^41^. The same applies for other PBUTs considered in this study, except percentage bound differs among toxins (Table 2). Albumin is found in the extracellular compartment (EC) only, hence it can be stated that distribution of strongly bound PBUTs, such as IS and pCS is limited to EC. Within EC, albumin is distributed in plasma and interstitial pool (~40% albumin mass in plasma), and thus albumin-bound toxins are assumed to distribute in plasma and interstitial pool, separated by the capillary endothelium. One should pay attention to the physiological aspect that the available free fraction can freely distribute in the intracellular compartment (IC) as well, like any other non-protein bound small size toxin. Distribution in IC will be more significant for moderately/weakly bound PBUTs such as IAA (~73% bound) and pCG (~15% bound). Whether this PBUT concentration in IC exist in free form only or in bound form as well, is not known clearly. *In vivo* distribution of hippuric acid and its derivative between red cells and plasma shows much higher concentration in plasma than in the cellular compartment^42^. This suggests that only free concentration may exist in the cellular pool, responsible for cytotoxicity and other side-effects, e.g., the intracellular accumulation of pCS has been implicated in vascular toxicity and smooth muscle cell damage^26^. Based on this, it is assumed that only the free fraction of PBUTs exists in IC, which remains in equilibrium with free PBUT concentration in interstitial compartment. Toxins are generated in IC and then diffuse to EC where they bind to albumin depending on individual toxin binding affinity determined by the equilibrium association constant (*K*_*A*,*toxin*_).

**Figure 8:**
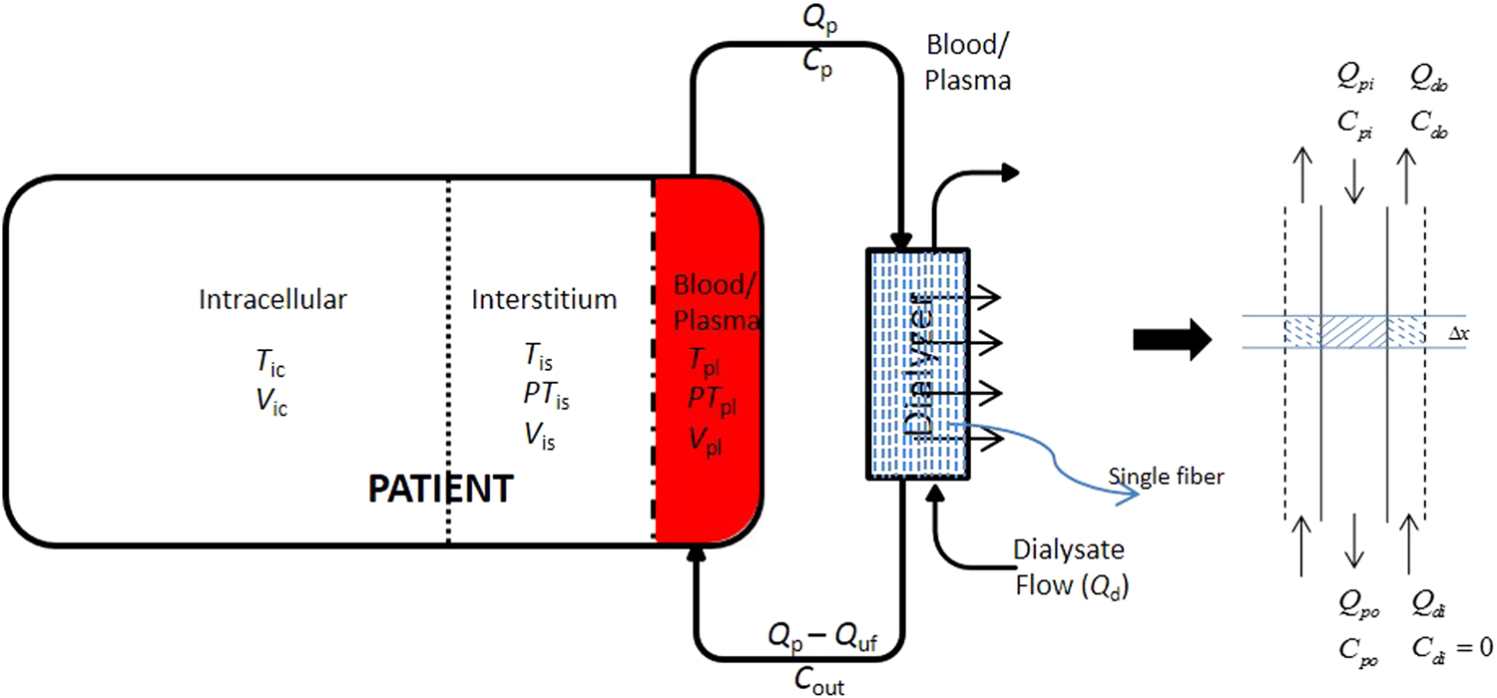
Block diagram of the model for protein-bound uremic toxin distribution and removal during dialysis. A three-compartmental model of the patient connected to dialyzer is shown. Here, *T*, *PT*, *V* denote free toxin concentration, protein-bound toxin concentration, and distribution volume in subscripted compartment plasma (pl), interstitial (is) and intracellular (ic); *Q*_p_, *Q*_d_, and *Q*_uf_ denote plasma flow rate, dialysate flow rate, and ultrafiltration rate, respectively; *C*_p_ denote free toxin or protein-bound toxin or toxin free protein concentration in dialyzer blood inlet and *C*_out_ in dialyzer blood outlet. A single dialyzer fiber is magnified to depict the counter-current blood and dialysate flow in dialyzer. A small fiber segment Δ*x* is shown through which free toxin transfers from the plasma side to the dialysate side. Here, *Q*_*pi*_ and *Q*_*di*_ denote inlet plasma and dialysate flow rate; *Q*_*po*_ and *Q*_*do*_ are outlet plasma and dialysate flow rates; *C*_*pi*_ and *C*_*di*_ are the molar concentrations at the plasma and dialysate inlet, respectively; *C*_*po*_ and *C*_*do*_ are the molar concentrations at the respective outlets.

The block diagram of the model is shown in Figure 8, where a three-compartmental representation of patient is connected to the dialyzer. Toxin laden blood leaves the patient and passes through the dialyzer. In the dialyzer, free toxin diffuses from the blood side to the dialysate side. A small fraction of free toxin will also leave with the ultra-filtered fluid (convective transfer). Downstream the dialyzer, “clean” blood goes back to patient, and the process continues for 4 hours in the most conventional HD settings.

Unlike other modeling approaches of PBUT kinetics where diffusive exchange between compartments was calculated based on total toxin concentration difference (sum of free and bound)^4^, the proposed model considers a more physiological scenario where only free toxin is exchanged between the plasma and interstitial compartment. It is assumed that albumin and the albumin-toxin complex do not exchange between plasma and interstitial compartments^43^. Dialytic removal of free toxin from the plasma pool leads to diffusive transfer of the free fraction from interstitial to plasma pool followed by toxin transfer from IC to interstitial pool. Reduced free toxin concentration in plasma and interstitial pool also drives the dissociation of protein-toxin complex in the respective compartments. It is assumed that approximately 40% of total albumin mass is distributed in plasma and 60% in interstitial pool, in agreement with albumin distribution in HD patients^44^. However, the interstitial fluid volume is much larger than that of plasma; conversely the albumin concentration in interstitial pool is much smaller than the plasma albumin concentration. The same analogy also applies to the albumin-toxin complex. In the model presented here, toxin mass balance in the plasma compartment can be written as,

$$\begin{array}{rcl}{\rm{Rate}}\,{\rm{of}}\,{\rm{toxin}}\,{\rm{accumulation}} & = & -\,{\rm{Toxin}}\,{\rm{leaving}}\,{\rm{plasma}}\,{\rm{compartment}}\,{\rm{with}}\,({Q}_{p})\\  &  & +\,{\rm{Toxin}}\,{\rm{entering}}\,{\rm{plasma}}\,{\rm{compartment}}\,{\rm{with}}\,({Q}_{p}\,-\,{Q}_{uf})\\  &  & +\,{\rm{Diffusive}}\,{\rm{mass}}\,{\rm{transfer}}\,{\rm{from}}\,{\rm{interstitial}}\,{\rm{space}}\\  &  & +\,{\rm{Convective}}\,{\rm{mass}}\,{\rm{transfer}}\,{\rm{from}}\,{\rm{interstitial}}\,{\rm{space}}\\  &  & +\,{\rm{Reaction}}\,{\rm{rate}}\,{\rm{terms}}\,{\rm{based}}\,{\rm{on}}\,\mathrm{first} \mbox{-} \mathrm{order}\,{\rm{kinetics}}\end{array}$$

where *Q*_*p*_ and *Q*_*uf*_ are the plasma flow rate and the ultrafiltration rate, respectively. The resulting mass balance equations for free toxin, the protein-toxin complex, and free protein (protein without toxin) in the plasma compartment are shown in Equation (1). The protein-toxin complex reaction kinetics is governed by the Law of Mass Action^45^.

1 $$\begin{array}{rcl}\frac{d({V}_{pl}{T}_{pl})}{dt} & = & -{Q}_{p}{T}_{pl}+({Q}_{p}-{Q}_{uf}){T}_{out}+{K}_{ip,T}({T}_{is}-{T}_{pl})+\alpha {Q}_{uf}{T}_{is}+(-{k}_{1}{P}_{pl}{T}_{pl}+{k}_{2}P{T}_{pl}){V}_{pl},\\ \frac{d({V}_{pl}P{T}_{pl})}{dt} & = & -{Q}_{p}P{T}_{pl}+({Q}_{p}-{Q}_{uf})P{T}_{out}+({k}_{1}{P}_{pl}{T}_{pl}-{k}_{2}P{T}_{pl}){V}_{pl},\\ \frac{d({V}_{pl}{P}_{pl})}{dt} & = & -{Q}_{p}{P}_{pl}+({Q}_{p}-{Q}_{uf}){P}_{out}+(-{k}_{1}{P}_{pl}{T}_{pl}+{k}_{2}P{T}_{pl}){V}_{pl}\end{array}$$

Similarly, mass balance for free toxin, the protein-toxin complex, and free protein in the interstitial compartment is given by Equation (2),

2 $$\begin{array}{rcl}\frac{d({V}_{is}{T}_{is})}{dt} & = & {K}_{ic,T}({T}_{ic}-{T}_{is})-{K}_{ip,T}({T}_{is}-{T}_{pl})-\alpha {Q}_{uf}{T}_{is}+(-{k}_{1}{P}_{is}{T}_{is}+{k}_{2}P{T}_{is}){V}_{is},\\ \frac{d({V}_{is}P{T}_{is})}{dt} & = & ({k}_{1}{P}_{is}{T}_{is}-{k}_{2}P{T}_{is}){V}_{is},\\ \frac{d({V}_{is}{P}_{is})}{dt} & = & (-{k}_{1}{P}_{is}{T}_{is}+{k}_{2}P{T}_{is}){V}_{is}\end{array}$$

and free toxin mass balance in the intracellular compartment is given by Equation (3), where toxin generation is assumed to occur at a constant rate and generated toxins diffuse to the interstitial compartment according to the concentration gradient,

3 $$\frac{d({V}_{ic}{T}_{ic})}{dt}=G-{K}_{ic,T}({T}_{ic}-{T}_{is})$$

During dialysis, a patient loses a significant amount of fluid. It is assumed that fluid is removed at a constant ultrafiltration rate (*Q*_*uf*_) and fluid removal by ultrafiltration occurs in proportion to the compartmental distribution volume^46^. Intracellular fluid volume is assumed constant during the course of dialysis, as observed by multi-frequency bioimpedance^47^. In the model, initial compartmental volumes are unknown model parameters and are estimated using toxin concentration data. Fluid balance for the plasma compartment is described as,

$$\begin{array}{rcl}{\rm{Rate}}\,{\rm{of}}\,{\rm{change}}\,{\rm{of}}\,{\rm{plasma}}\,{\rm{volume}} & = & {\rm{Fluid}}\,{\rm{leaving}}\,{\rm{the}}\,{\rm{patient}}\,(-{Q}_{p})\\  &  & +\,{\rm{Fluid}}\,{\rm{entering}}\,{\rm{the}}\,{\rm{patient}}\,({Q}_{p}-{Q}_{uf})\\  &  & +\,{\rm{Fluid}}\,{\rm{flow}}\,{\rm{from}}\,{\rm{interstitial}}\,{\rm{space}}\,(\alpha {Q}_{uf})\end{array}$$

Time dependent changes in plasma and interstitial fluid volumes are given by Equation (4).

4 $$\begin{array}{l}\frac{d({V}_{pl})}{dt}=-(1-\alpha ){Q}_{uf}\\ \begin{array}{cc}\frac{d({V}_{is})}{dt}=-\alpha {Q}_{uf} & where\,\alpha =\,\frac{{V}_{is}}{{V}_{is}+{V}_{pl}}\end{array}\end{array}$$

In Equations (1–4), *T*_pl_, *T*_is_, *T*_ic_, denote free toxin concentration in the plasma (pl), the interstitial (is), and the intracellular compartment (ic); *V*_*pl*_, *V*_*is*_, *V*_*ic*_ denote the respective compartmental fluid volumes; *PT*_*pl*_, *PT*_*is*_ are protein-toxin complex concentrations; *P*_*pl*_, *P*_*is*_ free protein concentrations in plasma and interstitial compartment; *G* is the toxin generation rate; *α* is the fraction of extracellular fluid volume in the interstitial compartment.

Of note, the present model assumes that there is one protein-toxin binding site. In reality, the majority of PBUTs have more than one binding site on the albumin molecule. For instance, IS primarily binds to Sudlow Site II in subdomain IIIA^45^. It also binds to a secondary, low-affinity binding site, however the majority of IS binds to the primary, high-affinity binding site^41^. As such, the equilibrium association constant used in the model represents the overall binding affinity for all albumin binding sites of any given PBUT. Also, the developed model does not consider competition among PBUTs, i.e., each PBUT is assumed to interact independently with albumin molecule in the plasma and interstitial pool, as well as in the dialyzer.

#### (B) *Dialyzer Model*

Blood/plasma and dialysate flow are counter-current. Free toxin diffuses from the blood to the dialysate side, resulting in dissociation of the protein-toxin complex and appearance of additional free toxin at the blood side. Hence, the conventional dialyzer clearance (*K*_D_) definition, which is calculated using the dialyzer inlet and outlet concentration and is defined for non-protein bound uremic solutes, is insufficient to describe the complex dynamics of PBUTs. In the model presented here, the membrane mass transfer-area coefficient (${{\mathscr{K}}}_{o}{\mathscr{A}}$) is used for calculating the diffusive mass transfer across hollow fibers. ${{\mathscr{K}}}_{o}{\mathscr{A}}$ is a membrane property and depends on the molecular weight of the toxin, but remains fairly constant below 500 Da^48^. The PBUTs considered in this study are much smaller in size (175–284 g/mol); hence, we assumed the same ${{\mathscr{K}}}_{o}{\mathscr{A}}$ for all PBUTs. A schematic of blood and dialysate flow across a fiber is shown in Figure 8. We assumed that blood flow is evenly distributed in all fibers; similarly dialysate flow is evenly distributed in the interstitial space between hollow fibers. In the model presented here, both blood and dialysate side mass balance equations are included.

#### Blood Side Model

The spatiotemporal depiction of concentration of toxin, the protein-toxin complex, and free protein at the blood side is shown by Equation (5).

5 $$\begin{array}{ccc}\frac{{\rm{\partial }}T}{{\rm{\partial }}t} & = & -\frac{1}{NA}\frac{{\rm{\partial }}}{{\rm{\partial }}x}({Q}_{p}T)-\frac{1}{NAL}({{\mathscr{K}}}_{{\mathscr{o}}}{\mathscr{A}}(T-{T}_{d})+{Q}_{uf}\bar{T})+(-{k}_{1}P\cdot T+{k}_{2}PT)\\ \frac{{\rm{\partial }}(PT)}{{\rm{\partial }}t} & = & -\frac{1}{NA}\frac{{\rm{\partial }}}{{\rm{\partial }}x}({Q}_{p}PT)+({k}_{1}P\cdot T-{k}_{2}PT)\\ \frac{{\rm{\partial }}P}{{\rm{\partial }}t} & = & -\frac{1}{NA}\frac{{\rm{\partial }}}{{\rm{\partial }}x}({Q}_{p}P)+(-{k}_{1}P\cdot T+{k}_{2}PT)\end{array}$$

Initial and boundary conditions for Equation (5) are,

$$\begin{array}{c}{{C}_{p}(x)|}_{t=0}=0,\\ {{C}_{p}(t)|}_{x=0}={\rm{Plasma}}\,{\rm{concentration}}\,{\rm{from}}\,{\rm{patient}},\,{\rm{where}}\,C=T\,or\,C=PT\,or\,C=P.\end{array}$$

Here, ${{\mathscr{K}}}_{o}{\mathscr{A}}(T-{T}_{d})+{Q}_{uf}\bar{T}$ denotes the diffusive and convective toxin transfer from the blood to the dialysate side. Toxins in the dialyzer are removed by diffusion as well as convection. However, the diffusive and convective removal contributions are not additive, for e.g. increased convection depletes the free concentration at the blood side, resulting in a smaller diffusive gradient, and vice versa. To account for diffusive and convective flux, the Péclet number $Pe=\frac{{Q}_{uf}}{{{\mathscr{K}}}_{o}{\mathscr{A}}}$ is used, which adjusts the convective flux in presence of a diffusive flux. Here, $\overline{T\,}$ denotes the free toxin concentration inside membrane contributing towards convective flux. It is a function of both, free toxin concentration at the plasma side *T* and at the dialysate side *T*_*d*_, $\overline{T\,}=T(1-\phi )+{T}_{d}\phi$, where $\phi =\frac{1}{Pe}-\frac{1}{{e}^{Pe}-1}$ ^49^. Considering uniform and equal blood flow across all fibers, it can be stated that diffusive removal by an individual fiber will be driven by $\frac{{{\mathscr{K}}}_{o}{\mathscr{A}}}{N}$, where *N* is the total number of fibers.

In the dialyzer, plasma flow rate (*Q*_*p*_) decreases along the fiber length due to ultrafiltration. It is assumed that *Q*_*p*_ decreases linearly along the fiber length (Equation (6)). In the current model, back filtration in the dialyzer is not considered.

6 $${Q}_{p}(x)={Q}_{pi}-\frac{x}{L}{Q}_{uf}$$

Here *Q*_*p*_(*x*) is the plasma flow rate at position *x* along the fiber length; *A* is the inner cross-sectional area of the fiber through which blood flows. The axial diffusion along the fiber length is neglected owing to a smaller axial diffusion coefficient for the solute in blood^50^. This is equivalent to plug flow assumption and there is no mass transfer between adjacent plugs.

#### Dialysate Side Model

Dialysate flows in the annulus space around each fiber. It is assumed that the dialysate flow is uniform and equally shared by the *N* fibers present in the dialyzer housing. Similar to the blood side model, the spatiotemporal description of the concentration within annulus dialysate flow boundary (shown by dashed lines around magnified fiber in Figure 8) is given by Equation (7).

7 $$\frac{{\rm{\partial }}{T}_{d}}{{\rm{\partial }}t}=\frac{1}{N{A}_{d}}\frac{{\rm{\partial }}}{{\rm{\partial }}x}({Q}_{d}{T}_{d})+\frac{1}{N{A}_{d}L}({{\mathscr{K}}}_{o}{\mathscr{A}}(T-{T}_{d})+{Q}_{uf}\bar{T})$$

Here, *A*_*d*_ is the dialysate flow area around a single fiber. If *D*_*h*_ is the diameter of the dialyzer housing encasing *N* fibers, *r*_*f*_ is the inner radius of the fiber, *t*_*f*_ the fiber thickness, then *A*_*d*_ can be calculated as shown in Equation (8).

8 $${A}_{d}=\frac{\pi {D}_{h}^{2}}{4N}-\pi {({r}_{f}+{t}_{f})}^{2}$$

Also, the dialysate flow increases by the amount of fluid removed by ultrafiltration from the blood side to the dialysate side, as shown in the Equation (9),

9 $${Q}_{d}(x)={Q}_{di}+\frac{L-x}{L}{Q}_{uf}$$

Here *Q*_*di*_ is the inlet dialysate flow rate, *L* is fiber length, and *x* is axial location in fiber. Only free toxin is considered for the dialysate side model (Equation (7)), as there is no free protein or protein-toxin complex at the dialysate side. Initial and boundary conditions for Equation (7) are,

$$\begin{array}{c}{{T}_{d}(x)|}_{t=0}=0\\ {{T}_{d}(t)|}_{x=L}=0\end{array}$$

Toxin transfer from the blood side to the dialysate side experiences three resistances namely, (1) the blood side boundary layer, (2) the hollow fiber membrane resistance, and (3) the dialysate side boundary layer. In the model presented here, the membrane mass transfer-area coefficient ${{\mathscr{K}}}_{o}{\mathscr{A}}$ or surrogate of membrane resistance lumps all three resistances together. It is also assumed that ${{\mathscr{K}}}_{o}{\mathscr{A}}$ remains constant during the course of dialysis, i.e., membrane pore clogging and changes in blood or dialysate flow rates do not affect ${{\mathscr{K}}}_{o}{\mathscr{A}}$.

### Model calibration

Starting concentrations and intra-dialytic concentrations of IAA, IS, pCG, pCS, and urea were extracted from Eloot *et al*. (for the one representative patient shown in that publication)^4^. To extract the concentration data from figures, we used WebPlotDigitizer^51^. The degree of protein binding for the individual toxins were obtained from the same paper (averages of 10 patients, Table 2). Similarly, average hematocrit of 37% for 10 patients was assigned as patient hematocrit; *Q*_b_ and *Q*_d_ were 300 mL/min and 700 mL/min, respectively; total ultrafiltration volume was 2.0 L in 4 hours of HD^4^, and ultrafiltration rate was kept constant. Pre-dialysis serum albumin concentration was assumed to be 4 g/dL. The patients in Eloot *et al*.^4^ had a median residual renal urea clearance of 2.6 mL/min, however we did not include its contribution towards PBUT removal during dialysis. The model calibration was performed using the F180NR dialyzer specifications: fiber count 12300, inner radius of single fiber (*r*_*f*_) 105 µm, fiber thickness (*t*_*f*_) 35 µm, fiber length (L) 23 cm, and dialyzer housing diameter (*D*_h_) 4 cm (personal communication with Fresenius Medical Care, Bad Homburg, Germany).

For the F180NR dialyzer used in the model simulations, the average *in vitro* ${{\mathscr{K}}}_{o}{\mathscr{A}}$ for IS, pCS, and Hippurate was 761 mL/min^12^. According to Hootkins *et al*.^52^, this *in vitro* ${{\mathscr{K}}}_{o}{\mathscr{A}}$ should be reduced by 20% when working with *in vivo* data, resulting in ${{\mathscr{K}}}_{o}{\mathscr{A}}$ of 609 mL/min. In the model simulations here, we used ${{\mathscr{K}}}_{o}{\mathscr{A}}$ of 600 mL/min. The equilibrium association constant (*K*_*A*,*toxin*_) for individual toxin was calculated as the ratio of pre-dialysis protein-toxin complex concentration (*PT*_0_) to the product of pre-dialysis free toxin concentration (*T*_0_) and free protein concentration (*P*_0_). The *K*_*A*,*toxin*_ is a characteristic binding property of a ligand to albumin, and it is expressed in Equation (10),

10 $${K}_{A,toxin}=\frac{{k}_{1}}{{k}_{2}}=\frac{P{T}_{0}}{{P}_{0}{T}_{0}}$$

with the above parameters specified, initial plasma volume (*V*_*pl0*_), initial extracellular fluid volume (*V*_*ex0*_), intra-cellular fluid volume (*V*_*ic*_), toxin mass transfer coefficient between plasma and interstitial pool (*K*_*ip*,*T*_), toxin mass transfer coefficient between interstitial and intracellular compartment (*K*_*ic*,*T*_), and the association constant (*k*_1_) for individual toxin in the dynamic equilibrium $P+T\,\rightleftharpoons PT$ were estimated for the representative patient published by Eloot *et al*.^4^. The two-pool urea kinetics model was also calibrated using urea concentration data to estimate urea mass transfer coefficient (*K*_ic,T_). The compartmental volumes *V*_*pl0*_, *V*_*ex0*_ and *V*_*ic*_ were jointly optimized across all toxins. The model has one more unknown parameter – the dissociation constant (*k*_2_) of the protein-toxin complex. It is obtained using the definition of the *K*_*A*,*toxin*_, with *k*_1_ estimated using patient data.

We also calculated the individual toxin generation rate for the given parameter vector such that the pre-dialysis concentration at the next session is 95% of pre-dialysis concentration at the current session. We used 95% as reference because the toxin concentration data comes from a mid-week session and next HD session will be the last session of the week, in which pre-dialysis concentration will be smaller than preceding session of the week. In the calculation of generation rate, it is assumed that patient ingest 2 L of fluid in inter-dialytic interval (same as the amount removed during dialysis). We repeated the generation rate calculation followed by model calibration, until there is negligible change in individual toxin generation rate. Model fit was quantified by relative error per toxin defined as,

$$Relative\,Error=\sqrt{\sum _{i}{(\frac{{y}_{pred,i}-{y}_{obs,i}}{{y}_{obs,i}})}^{2}};i=0,15,30,60,120,240\,min$$

### Model validation

We first compared model simulation results with *in vivo* data of Deltombe *et al*., who studied 10 HD patients (demographics reproduced in Table 1)^23^. To test the model performance against these *in vivo* results, the initial PBUT concentrations for model simulations were adopted from the model calibration step (see Figure 1)^4^. The model parameters identified during model calibration (Table 3) were likewise adopted for these simulations. We tested the model performance on three domains as stated in the Results section. For the first two domains, the initial degree of protein binding was varied to cover the ranges seen in the 10 patients (Table 4), as reported by Deltombe *et al*.^23^. For the third domain, we kept the initial protein-binding equal to the one used in model calibration step (Table 2). Note that the comparison of simulation data with experimental data is only done in aggregate (mean ± SD). This is due to the fact that, in Deltombe *et al*.^23^, protein binding data are only provided in aggregated form (no time curves for individual patients). It is therefore impossible to evaluate whether, on an individual level, the simulated data are well correlated to the experimental data.

Second, we compared the model performance against *in vitro* data of Meyer *et al*. who studied the effect of increase in the dialysate flow (*Q*_d_) and dialyzer mass transfer-area coefficient (${{\mathscr{K}}}_{o}{\mathscr{A}}$)^1^. Meyer *et al*. studied the two dialyzers F6 and F200NR. In our model calibration step, we used the F180NR dialyzer. To account for different dialyzer specification of Meyer *et al*., we decreased and increased the number of fibers for F6 and F200NR, respectively. As mentioned earlier, the F180NR dialyzer with membrane surface area of 1.8 m^2^ comprises 12300 fibers, which were reduced to 8800 for the F6 dialyzer to obtain a surface area of 1.3 m^2^, and increased to 13660 for F200NR with a surface area of 2.0 m^2^. Other fiber characteristics, namely were kept the same. To simulate the actual *in vitro* setup comprising a plasma pool volume of 4.5 L connected to the dialyzer, the developed model was simplified by neglecting interstitial and intracellular compartments in the patient model described earlier. Meyer *et al*. described a clearance equation, which accounts for a toxin free fraction. We used this equation to calculate ${{\mathscr{K}}}_{o}{\mathscr{A}}$ using *in vitro* phenol red clearance (*Cl*_*PR*_) given for each combination of dialysate flow rate and dialyzer mass transfer-area coefficient^1^. The clearance equation is reproduced here in Equation (11).

11 $$C{l}_{PR}={Q}_{p}(1-\frac{f-\theta }{{\rm{\Theta }}f-\theta }),where,{\rm{\Theta }}={e}^{{{\mathscr{K}}}_{{\mathscr{o}}}{\mathscr{A}}(\frac{f}{{Q}_{p}}-\frac{1}{{Q}_{d}})}\,and,\theta =\frac{{Q}_{p}}{{Q}_{d}}$$

other experimental characteristics were also emulated for model simulation, namely, phenol red initial free fraction 6% with equilibrium association constant 2.8 × 10^4^ M^−1^, experiment duration 120 min. For the association constant (*k*_1_) of phenol red, we used the one estimated for pCS (Table 3), since pCS and phenol red have similar initial bound fractions.

### Model predictions

We employed the calibrated model to characterize the compartmental distribution of all PBUTs. Corresponding results are presented in Figure 6. Note that we presented the concentration profiles scaled with respect to the initial total concentration (top panels) and the initial free concentration (bottom panels). This presentation of PBUT concentrations clearly delineates the unequal molar distribution of toxins in plasma and interstitial compartment due to disparate albumin distribution. Also, scaling allows for side-by-side comparison among PBUTs. Further, we used the calibrated model to study the effect of increasing blood and dialysate flow rates. The blood flow rate was increased between 300 and 500 mL/min, while the dialysate flow rate increased from 500 to 800 mL/min, both in steps of 10 mL/min. Dialyzer characteristics and individual toxin parameters were adopted from the model calibration step. The RRs were plotted in Figure 7. All model calibration, validation, and additional simulations were performed in MATLAB 2016a and R 3.2.3.

## Electronic supplementary material

Supplemental Material to “A novel mathematical model of protein-bound uremic toxin kinetics during hemodialysis”
