# Supplementary material for: A novel mathematical model of protein-bound uremic toxin kinetics during hemodialysis
Source: Sci Rep. 2017 Sep 4;7:10371. doi: 10.1038/s41598-017-10981-z (PMC5583320; doi:10.1038/s41598-017-10981-z)
Supplement: Supplementary file 1 — Supplemental Material to “A novel mathematical model of protein-bound uremic toxin kinetics during hemodialysis” [file 41598_2017_10981_MOESM1_ESM.doc]

**Supplemental Material to “A novel mathematical model of protein-bound uremic toxin kinetics during hemodialysis”**

Vaibhav Maheshwari^1*^, Stephan Thijssen^1^, Xia Tao^1^, Doris Fuertinger^1^, Franz Kappel^2^, Peter Kotanko^1,3^

^1^Renal Research Institute, New York, USA

^2^Institute for Mathematics and Scientific Computing, University of Graz, Austria

^3^Icahn School of Medicine at Mount Sinai, New York, USA

One of the interesting findings of our work pertains to the effect of blood and dialysate flow, which states that increase in dialysate flow improves the removal of strongly bound PBUTs whereas for the removal of less strongly bound PBUTs, one needs to increase the blood flow rate. Below we have provided a physical explanation for this phenomenon.

Effect of blood flow rate (*Q*_b_): In the case of weakly bound toxins (or unbound molecules like urea or creatinine), free toxin concentration is high in blood/plasma. When this free toxin laden blood passes through the dialyzer, a large fraction of this free toxin is removed in a single pass. After a single pass, the toxin concentration at the dialyzer outlet will be very small. We tested this scenario for a standalone dialyzer model and calculated the reduction ratio across the dialyzer (Figure S1). Note that under these conditions the reduction ratio for IS and pCS is much smaller compared to IAA and pCG. When venous (outlet) blood from the dialyzer mixes with systemic blood, the toxin concentration in the plasma drops. Increasing the blood flow rate increases the rate of blood circulating through the dialyzer and as such the solute removal across the dialyzer. Since IAA and pCG experience greater removal across the dialyzer, increasing the blood flow will lead to a pronounced drop in their concentrations, resulting in a greater reduction ratio during the course of dialysis. However, the situation is very different for strongly bound PBUTs. In the case of IS and pCS, owing to strong protein-binding, the dialyzer outlet concentrations are reduced only slightly. Mixing of this dialyzer outlet blood with blood in the systemic circulation will result in only a very small decrease in their concentrations. Hence, increasing the blood flow rate (i.e. reducing blood recirculation time) will have only a minor effect on the RR of strongly bound toxins.

**Figure S1**: Effect of blood and dialysate flow rate on toxin reduction ratio across the dialyzer. The concentrations of toxin and protein were kept constant at the dialyzer blood inlet for all simulations.

Figure S1 shows that an increase in blood flow rate reduces the RR across the dialyzer. This is due to reduced residence time of the blood in the dialyzer. Note that this finding is separate from what is presented in the manuscript (Figure 7), where we show the toxin reduction ratio in the systemic circulation over time.

Effect of dialysate flow rate (*Q*_d_): A higher dialysate flow rate will keep dialysate toxin concentrations lower and thereby increase the concentration gradient between blood and dialysate, resulting in greater solute removal. However, one may notice (Figure 7 in the manuscript) that dialysate flow apparently has a more pronounced effect on strongly bound toxins when compared to weakly bound toxins. As dialysate only affects removal along the dialyzer fiber, we simulated a scenario where we looked at toxin RR along the fiber with varying dialysate flow rates (Figure S2). Here, the *Q*_b_ was kept constant at 300 mL/min, and no ultrafiltration was applied. Note that, for any position along the dialyzer fiber, the RR of weakly bound or strongly bound toxins only marginally changes with dialysate flow rate, while the magnitude of RR for weakly bound toxins is much bigger than that of strongly bound toxins. Also, along the fiber, the RR of weakly bound toxins shows some saturation, while the RR of strongly bound toxins shows an upward trend.

**Figure S2**: Toxin reduction ratio along the dialyzer fiber as a function of dialysate flow rate.

In summary, the effect of blood flow rate significantly surpasses that of dialysate flow rate with respect to the clearance of weekly bound toxins. For strongly bound toxins, an increase in blood flow rate has a negligible impact on their RR, while an increase in dialysate flow rate as a more prominent effect.
